# Interpreter Child Syndrome Leading to Parentification and Anxiety in a Refugee Girl: A Case Report

**DOI:** 10.7759/cureus.99841

**Published:** 2025-12-22

**Authors:** Elanur Yolal Karimov

**Affiliations:** 1 Department of Pediatrics, Istanbul Kanuni Sultan Suleyman Training and Research Hospital, Social Pediatrics Doctorate Program, Istanbul Faculty of Medicine, Institute of of Health Sciences, Istanbul University, Istanbul, TUR

**Keywords:** adjustment disorder, anxiety in children, interpreter child, language brokering, parentification, refugee children, secondary traumatization

## Abstract

Language brokering, in which children interpret for non-native-speaking parents, is common among refugee families but is often overlooked as a potential psychosocial stressor. Studies indicate that 75-90% of immigrant and refugee children serve as language brokers, with 18-20% experiencing clinically significant psychological distress related to this role. Excessive or emotionally charged interpreting may lead to parentification, anxiety, and developmental disruption.

We present a case of a nine-year-old Syrian refugee girl who developed anxiety, somatic symptoms, academic decline, and emotional over-responsibility after serving as her mother's primary interpreter for nearly all daily interactions, including sensitive gynecological and mental health appointments. Her interpreting load averaged 10-15 h per week, resulting in role reversal, secondary traumatization, and premature emotional maturity characterized by excessive worry about adult matters, suppression of age-appropriate needs, and assuming responsibility for parental emotional well-being. Psychological evaluation showed moderate anxiety (Pediatric Anxiety Rating Scale {PARS} 18/25, clinical threshold ≥11) and borderline emotional difficulties (Strengths and Difficulties Questionnaire {SDQ} total 16, clinical range ≥17). She was diagnosed with adjustment disorder with mixed anxiety and depressed mood. Intervention included psychoeducation, restructuring family boundaries, prohibition of child interpreter use in medical settings, referral to professional interpreter services, and cognitive-behavioral/play therapy. Cognitive behavioral therapy (CBT) consisted of the following 12 weekly 50-minute sessions: sessions one to three focused on psychoeducation and rapport-building, sessions four to eight addressed cognitive restructuring, and sessions nine to 12 implemented behavioral strategies. Four family therapy sessions were conducted at weeks two, six, 10, and 14. Over six months, her anxiety, somatic complaints, and academic functioning improved significantly. While this single case cannot establish causation or prevalence, it suggests a potentially important clinical phenomenon requiring further investigation. The case highlights the psychological risks of using minors as interpreters in healthcare settings and suggests the importance of routine screening for language brokering roles among refugee children. Access to professional interpreter services may be essential to protect child wellbeing and maintain appropriate parent-child boundaries.

## Introduction

Turkey currently hosts over 3.6 million Syrian refugees, making it the largest refugee-hosting country in the world. Approximately 1.7 million of these refugees are children who face unique psychosocial challenges, such as trauma exposure, acculturation stress, and disrupted education [1]. While extensive research has examined post-traumatic stress and adaptation difficulties among refugee children, the phenomenon of language brokering, where children translate for their non-native-speaking parents, remains an underrecognized source of psychological burden within refugee populations [2].

Language brokering affects approximately 75-90% of immigrant and refugee children globally, with studies indicating that 18-20% experience clinically significant psychological distress related to this role [2]. Among Syrian refugee populations specifically, preliminary data suggest that up to 85% of children serve as family interpreters, with higher rates observed in families with limited access to professional interpretation services [3].

Language brokering is nearly universal among refugee families, as children typically acquire the host country's language more rapidly than their parents. Although this role may appear benign or even beneficial for language development, research indicates that frequent and emotionally intense language brokering can trigger parentification, a role reversal in which children assume adult responsibilities that exceed their developmental capacity [2,3].

Parentification has been linked to anxiety, depression, academic difficulties, and disturbances in identity formation. When children translate sensitive adult content, such as medical information, financial matters, or traumatic narratives, they are at risk of secondary traumatization and premature loss of childhood [4,5]. Despite its prevalence, this phenomenon remains underrepresented in clinical literature, particularly among Middle Eastern refugee populations [6,7].

This paper presents the case of a Syrian refugee child who developed significant psychological symptoms related to chronic language brokering and parentification, aiming to highlight the need for healthcare systems to recognize and address this overlooked issue.

## Case presentation

A nine-year-old Syrian girl, referred to as Amira (pseudonym), was brought to the pediatric outpatient clinic due to declining academic performance, frequent absences, and increased anxiety over the past six months. The family had lived in Istanbul for three years after being displaced from Aleppo in 2021 (specific date removed for confidentiality). Amira's symptoms included recurrent headaches, abdominal pain without medical cause, social withdrawal, irritability, and sleep disturbances. A detailed assessment revealed the following symptoms: headaches were tension-type, occurring three to four times weekly, lasting 2-3 h, often following translation tasks. Abdominal pain was periumbilical, non-radiating, without associated GI symptoms (negative medical workup including CBC, ESR, abdominal ultrasound). Sleep disturbances included delayed sleep onset (>45 min), frequent awakening (two to three times/night), with worry about family matters. Social withdrawal manifested as declined participation in previously enjoyed activities and reduced peer interactions from daily to once weekly.

Teachers described her as "acting like a little adult," specifically noting that Amira would intervene in peer conflicts as a mediator, expressed worry about classroom resources and teacher well-being, and frequently asked about administrative matters typically of no interest to nine-year-olds. Her developmental history was normal, and there was no significant medical or trauma history.

During psychosocial assessment, it was revealed that Amira had been serving as her mother's interpreter for three years in nearly all daily contexts. She accompanied her mother to medical appointments, including gynecological and mental health visits, translated at school meetings, municipal services, and asylum interviews, and handled communication in commercial and social settings. The estimated interpreting load was 10-15 h weekly, leading to missed school activities and emotional fatigue. Emotional fatigue was assessed as follows: (1) Pediatric Quality of Life Inventory (PedsQL) Multidimensional Fatigue Scale showing scores >2 SD below normative means, (2) structured interviews revealing decreased emotional availability after translation tasks, (3) activity logs showing reduced engagement in play following interpretation duties, and (4)​​​​​ observable behavioral changes including affective flattening during discussion of translation responsibilities.

On mental status examination, Amira appeared mature for her age, with anxious affect and excessive concern for her mother's reactions. Psychological testing showed moderate anxiety (Pediatric Anxiety Rating Scale {PARS} 18/25, clinical threshold ≥11) and borderline emotional difficulties (Strengths and Difficulties Questionnaire {SDQ} total 16, clinical range ≥17). Additional assessment with the screen for child anxiety-related disorders (SCARED) showed elevation only in situational anxiety subscales, not generalized anxiety. Physical examination was unremarkable except for mild muscle tension.

She was diagnosed with adjustment disorder with mixed anxiety and depressed mood (International Classification of Diseases {ICD}-10: F43.23), related to chronic role stress and parentification. Differential diagnoses, such as generalized anxiety disorder (GAD) were ruled out based on (1) detailed clinical interviews revealing anxiety symptoms were specifically triggered by translation situations rather than generalized worry across multiple domains, (2) use of SCARED showing elevation only in situational anxiety subscales, (3) temporal association between symptom onset and assumption of interpreter role, (4) absence of excessive worry about multiple life areas typical of GAD, and (5) symptoms improved with reduction in translation duties without anxiolytic medication. Post-traumatic stress disorder (PTSD) was ruled out due to the absence of direct trauma exposure.

Intervention included psychoeducation for the family, boundary setting prohibiting Amira from attending adult medical visits, and referral to professional interpreter services. Amira received 12 weekly 50-min cognitive-behavioral therapy sessions combined with play therapy elements. Sessions one to three were focused on psychoeducation about anxiety and establishing therapeutic rapport. Sessions four to eight addressed cognitive restructuring of excessive responsibility beliefs and catastrophic thinking patterns related to translation errors. Sessions nine to 12 implemented behavioral strategies, including assertiveness training, boundary-setting role-plays, and gradual exposure to age-appropriate independence. Family therapy consisted of four sessions conducted at weeks two, six, 10, and 14, targeting role restructuring and improving parent-child boundaries. School collaboration ensured academic support and prevention of further translation duties.

At three months, her school attendance improved from 60% to 85% and her grades improved from below average to average, somatic symptoms diminished (headache frequency reduced from three to four times weekly to once weekly), and anxiety scores decreased (PARS from 18 to 12) (Table 1). By six months, Amira exhibited normalized mood and behavior, resumed age-appropriate activities, and family boundaries improved with a PARS score of 8 (subclinical range) and an SDQ total score of 11 (normal range) (Table 1). Persistent challenges included occasional relapse during family stress and cultural expectations of child assistance.

**Table 1:** Assessment scores over treatment period. PARS: Pediatric Anxiety Rating Scale; SDQ: Strengths and Difficulties Questionnaire; PedsQL: Pediatric Quality of Life Inventory

Measures	Baseline	3 Months	6 Months	Clinical threshold
PARS (anxiety)	18	12	8	≥11
SDQ total	16	13	11	≥17
PedsQL fatigue	45	62	75	<60 (impaired)
School attendance (%)	60	85	92	-
Translation h/week	10-15	2-3	0-1	-

## Discussion

This case illustrates the phenomenon we term "interpreter child syndrome," referring to the cluster of psychological difficulties arising from chronic and developmentally inappropriate translation responsibilities among refugee children. The term "interpreter child syndrome" is used here as a non-diagnostic, descriptive concept to organize observed clinical features rather than to propose a new psychiatric diagnosis. Language brokering may be widespread among refugee families and may, under certain conditions, enhance linguistic competence and strengthen family cohesion [8]. However, this case suggests that when such translation duties become frequent, intense, and emotionally charged, they may contribute to clinically significant parentification, whereby children assume adult-like responsibilities that exceed their developmental capacity [2].

In refugee contexts, parentification appears to manifest in a culturally specific way. Although parentification has traditionally been documented in families affected by chronic illness, addiction, or mental health disorders, its expression through language brokering may constitute a distinct cultural variant [3]. In many refugee families, a child's role as a translator is perceived as a sign of maturity, responsibility, and devotion, and is often encouraged by parents [9]. This cultural acceptance may produce a psychological double bind as follows: children gain recognition within the family while potentially experiencing stress, emotional exhaustion, and role confusion.

Existing research on language brokering presents mixed findings [10]. Occasional, low-stakes interpreting has been associated with positive outcomes, such as cross-cultural adaptation, language enrichment, and stronger familial ties. However, when translation becomes frequent and emotionally laden, particularly in medical, legal, or financial contexts, exposure to adult themes and disruption of the child's developmental trajectory may transform the experience into a pathological one [11]. Amira's case exemplifies what may be this high-burden scenario as follows: her sustained interpreting role appeared to correlate with anxiety, somatization, and identity confusion.

One of the notable features of this case was potential secondary traumatization. Amira was repeatedly exposed to her mother's sensitive health information, including gynecological and psychological issues. While explicit traumatic narratives were not present in this case, the cumulative exposure to adult content beyond her cognitive capacity may have contributed to a form of vicarious stress. The literature supports this concept, with studies documenting increased anxiety and depression among child language brokers exposed to parental medical problems and asylum proceedings [12].

Healthcare providers' reliance on Amira as an interpreter, despite ethical guidelines against using minors for this purpose, highlights a systemic failure in refugee healthcare provision. International standards explicitly recommend against using children as interpreters in medical settings due to risks of psychological harm, miscommunication, and breach of parental confidentiality [13,14]. This case suggests that mere availability of professional interpreters is insufficient if healthcare workers are not trained to recognize and prevent child language brokering.

The apparent successful intervention in this case combined individual, family, and systemic approaches. The provision of professional interpreter services was particularly important, as it removed an immediate stressor while respecting the family's communication needs. Cognitive-behavioral therapy appeared to help Amira process her experiences and develop age-appropriate coping strategies, while family therapy seemed to address the underlying role reversal. However, the persistence of some symptoms during family stress suggests that long-term support may be necessary.

Cultural factors played a complex role in this case. The mother's initial resistance to professional interpreters reflected both practical concerns (availability and trust) and cultural values around family privacy and cohesion. In many Middle Eastern cultures, family matters are considered private, and involving outsiders, even professional interpreters, may be viewed as shameful or threatening [15,16]. The intervention's success required culturally sensitive negotiation, emphasizing that accepting help was a sign of good parenting rather than a sign of weakness.

This report has several important limitations. As a single case study, generalizability is limited, and causal relationships cannot be established. Multiple simultaneous interventions preclude clear attribution of effects to specific treatment components. The six-month follow-up period is insufficient to evaluate long-term outcomes. Potential confounders, including ongoing resettlement stress, parental mental health (mother's untreated depression), and school adjustment factors, were not fully controlled. Limited physiological data (e.g., cortisol sampling) were available to support stress-related hypotheses. Cultural and contextual factors specific to Istanbul may affect the transferability of findings to other settings. Nonetheless, this case provides valuable clinical insights that are rarely captured in large-scale studies, emphasizing the importance of systemic awareness, cultural sensitivity, and sustainable interpreter support in refugee care.

## Conclusions

While this single case cannot establish causation or prevalence, this case of "interpreter child syndrome" suggests that language brokering, while seemingly benign and often appreciated by families, may constitute a form of parentification with potential psychological consequences. Healthcare providers should consider that routinely using child interpreters, even when children and parents are willing, may represent an inadvertent violation of child well-being that could potentially impair development. The improvements observed in this case suggest the potential importance of (1) proactive screening for language-brokering roles; (2) providing psychoeducation about parentification in a culturally sensitive manner; (3) ensuring access to practical alternative resources, such as professional interpreters; (4) offering individual and family therapy to support role restructuring; (5) collaborating with schools to safeguard the child’s developmental and educational priorities; and (6) engaging in systemic advocacy to improve refugee families’ access to professional interpretation services.

As refugee populations continue to grow globally, healthcare systems should consider prioritizing professional interpreter services not merely as a best practice but as an ethical obligation to protect vulnerable children from potential psychological harm. This case adds to clinical practice by (1) providing a preliminary framework for identifying potential parentification in refugee children; (2) suggesting that early intervention may reverse associated symptoms; (3) offering a potentially replicable treatment approach that integrates individual-, family-, and systems-level interventions; and (4) highlighting the responsibility of healthcare providers to consider the psychological impact of inappropriate interpreter practices. The clinical bottom line is that healthcare providers should carefully consider the potential impact of using children as interpreters in healthcare settings, even when professional services are unavailable, as the potential short-term communication benefits may not justify the possible long-term psychological impact on the child.
